# Triboelectric Nanogenerator versus Piezoelectric Generator at Low Frequency (<4 Hz): A Quantitative Comparison

**DOI:** 10.1016/j.isci.2020.101286

**Published:** 2020-06-20

**Authors:** Abdelsalam Ahmed, Islam Hassan, Ahmed S. Helal, Vitor Sencadas, Ali Radhi, Chang Kyu Jeong, Maher F. El-Kady

**Affiliations:** 1 Department of Mechanical Engineering, Massachusetts Institute of Technology, Cambridge, MA, USA; 2 Division of Gastroenterology, Brigham and Women's Hospital, Harvard Medical School, Boston, MA, USA; 3 Department of Mechanical Engineering, McMaster University, Hamilton, ON, Canada; 4 Department of Nuclear Science and Engineering, Massachusetts Institute of Technology, Cambridge, MA, USA; 5 School of Mechanical, Materials, Mechatronic and Biomedical Engineering, University of Wollongong, Wollongong, NSW 2500, Australia; 6 ARC Centre of Excellence for Electromaterials Science, University of Wollongong, Wollongong, NSW 2500, Australia; 7 Department of Mechanical and Industrial Engineering, University of Toronto, Toronto, ON, Canada; 8 Division of Advanced Materials Engineering, Jeonbuk National University, Jeonju, Jeonbuk 54896, Republic of Korea; 9 Hydrogen and Fuel Cell Research Center, Jeonbuk National University, Jeonju, Jeonbuk 54896, Republic of Korea; 10 Department of Chemistry and Biochemistry and California NanoSystems Institute, University of California, Los Angeles (UCLA), Loss Angeles, CA, USA; 11 Department of Materials Science and Engineering, UCLA, Los Angeles, CA, USA

**Keywords:** Nanotechnology, Energy Resources, Energy Application, Nanomaterials

## Abstract

Triboelectric nanogenerators (TENGs) and piezoelectric generators (PGs) are generally considered the two most common approaches for harvesting ambient mechanical energy that is ubiquitous in our everyday life. The main difference between the two generators lies in their respective working frequency range. Despite the remarkable progress, there has been no quantitative studies on the operating frequency band of the two generators at frequency values below 4 Hz, typical of human motion. Here, the two generators are systematically compared based on their energy harvesting capabilities below 4 Hz. Unlike PGs, the TENG demonstrates higher power performance and is almost independent of the operating frequency, making it highly efficient for multi-frequency operation. In addition, PGs were shown to be inapplicable for charging capacitors when a rectifier was attached to the system. The results of this work reveal the tremendous potential of flexible TENGs for harvesting energy at low frequency.

## Highlights

• This work presents a systematic comparative study between PGs and TENGs below 4 Hz
• Unlike PGs, the TENG demonstrates higher power performance at low-frequency range
• Also, PGs was shown to be inapplicable for charging capacitors when a rectifier was used
• This work reveals the tremendous potential of TENGs as an energy harvester

## Graphical Abstract

**Figure:**
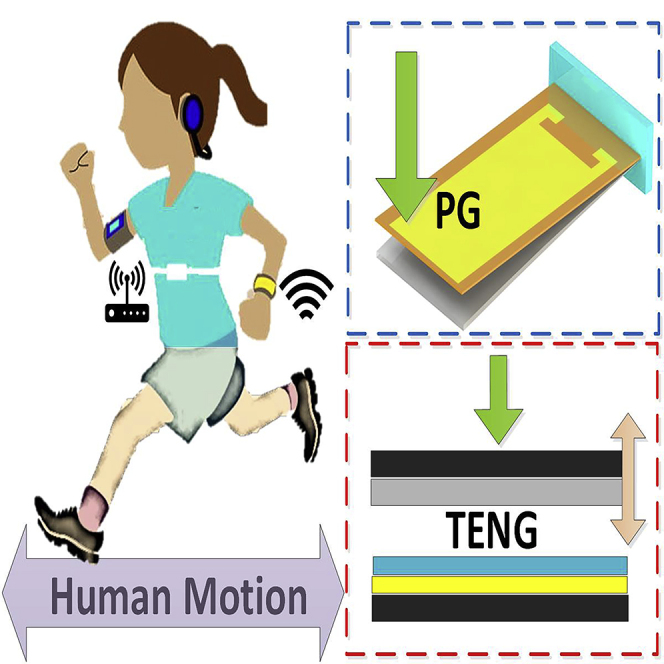

## Introduction

Small-scale electronics has boomed during the last decade for the Internet of Things (IoT), wireless sensor networks (WSNs), smart city design, and medical application (Gubbi et al., 2013; Jaladi et al., 2017). The components of such devices typically require a minimal amount of power, but it becomes a challenge when considering a large network of devices reaching up to billions. Moreover, maintenance cost may explode as the number of distributed micro-electronics increases when considering existing battery-based electronics, where their batteries are prone to replacements after its service life. The field of energy harvesting tries to answer these challenges by developing energy conversion schemes from ambient energy existing in the ecosystem such that electrical output is generated to allow for self-powered electronics (Mateu et al., 2005; Elvin and Erturk, 2013). In particular, mechanical energy is known for its abundance in the surrounding environment, readily available for potential energy harvesting apparatus based on electromagnetic (Yang et al., 2009), piezoelectric (Kim et al., 2011; Wang, 2008), triboelectric (Wang, 2013; Ahmed et al., 2019b) (Ahmed et al., 2020a), or electrostatic (Boisseau et al., 2012). Of these approaches, triboelectric nanogenerators (TENGs) and piezoelectric generators (PGs) have more significant potential for applications and were hybridized and coupled on numerous occasions during the past few years (Ahmed et al., 2017b, Ahmed et al., 2017; Chen et al., 2017).

The available mechanical energy found in the environment typically experiences low-frequency behaviors (around 10 Hz and lower). Examples of such mechanical motion are abundantly found in oceanic waves, human motion, wind currents, and other wildlife species (Naruse et al., 2009; Salauddin and Park, 2017). Harvesting such low-frequency sources may not be the best when utilizing piezoelectric technologies because PGs are known to be efficient at higher frequency ranges (60–100 Hz and above) (Liu et al., 2018; Han et al., 2013). The recent research effort has been dedicated to broadening the operating frequency range of PGs to include low-frequency loads (Wang, 2017; Jeong et al., 2017a). However, there are significant obstacles that still pose a challenge for PGs to reach such extremely low levels of mechanical frequencies (<10 Hz or sub 1 Hz frequencies). On the other hand, TENGs, owing to their attractive attributes, have been successfully applied for harvesting all kinds of mechanical energy such as vibration, human body motion, animal organs wind power, and water motion (Yang et al., 2013; Ahmed et al., 2017a, 2017c, 2017d, Ahmed et al., 2017, 2017f, Ahmed et al., 2018, 2019a, 2019c, 2019d, 2020b). A theoretical comparison between TENGs and PGs was previously conducted and reported by Han et al., 2015. However, a quantitative comparison has not been conducted yet regarding the operating frequency band between the two generators from low-frequency mechanical excitations.

Here, we present a comparative study and analysis between TENGs and PGs on low-frequency mechanical energy harvesting. In order to achieve a more systematic comparison, a simplified design of the TENG is compared with a chosen commercial PG. Contact separation (CS) TENG was fabricated, whereas the PG was made to operate in similar operating mode (Impact mode “IM”). For both energy harvesters, each mode is investigated and their respective open-circuit voltage ${\text{V}}_{\text{OC}}$ and short-circuit current ${\text{I}}_{\text{SC}}$ are used for electrical output comparison at multiple frequency values <4 Hz. PGs are efficient at much higher frequency values, where their extremely small outputs limit their uses for powering small-electronics or charging capacitors. TENG will be shown to have a maintainable and sustainable voltage and energy values, regardless of the frequency of mechanical excitation. This study shows the high efficiency of TENGS over PGs at a frequency less than 4 Hz for harvesting low-frequency mechanical loads.

## Results

This study aims to compare two of the most promising energy harvesting technologies, namely, TENGs and PGs, where they share several attractive features such as relatively low costs, ease of fabrication, and integrability in flexible electronics. CS operating mode is widely used in mechanical energy harvesting owing to their simplicity, efficiency, and compatibility with multi-directional loads (Yang et al., 2013). This mode was carefully selected as our criteria of comparison because it can be applied to both TENGs and PGs at the same time. Moreover, the CS mode was previously coupled in various device configurations owing to their outstanding performance in low-frequency/amplitude excitations. Analogous to TENGs, PGs were set up to have similar operating modes. The set up with CS mode was made possible by exerting an impact load on the PG (IM mode) in a contact-separation manner (Fuh et al., 2015; Gu, 2011). Therefore, this study utilizes the proposed modes as a foundation of systematic comparison between the two generators.

**Figure 1:**
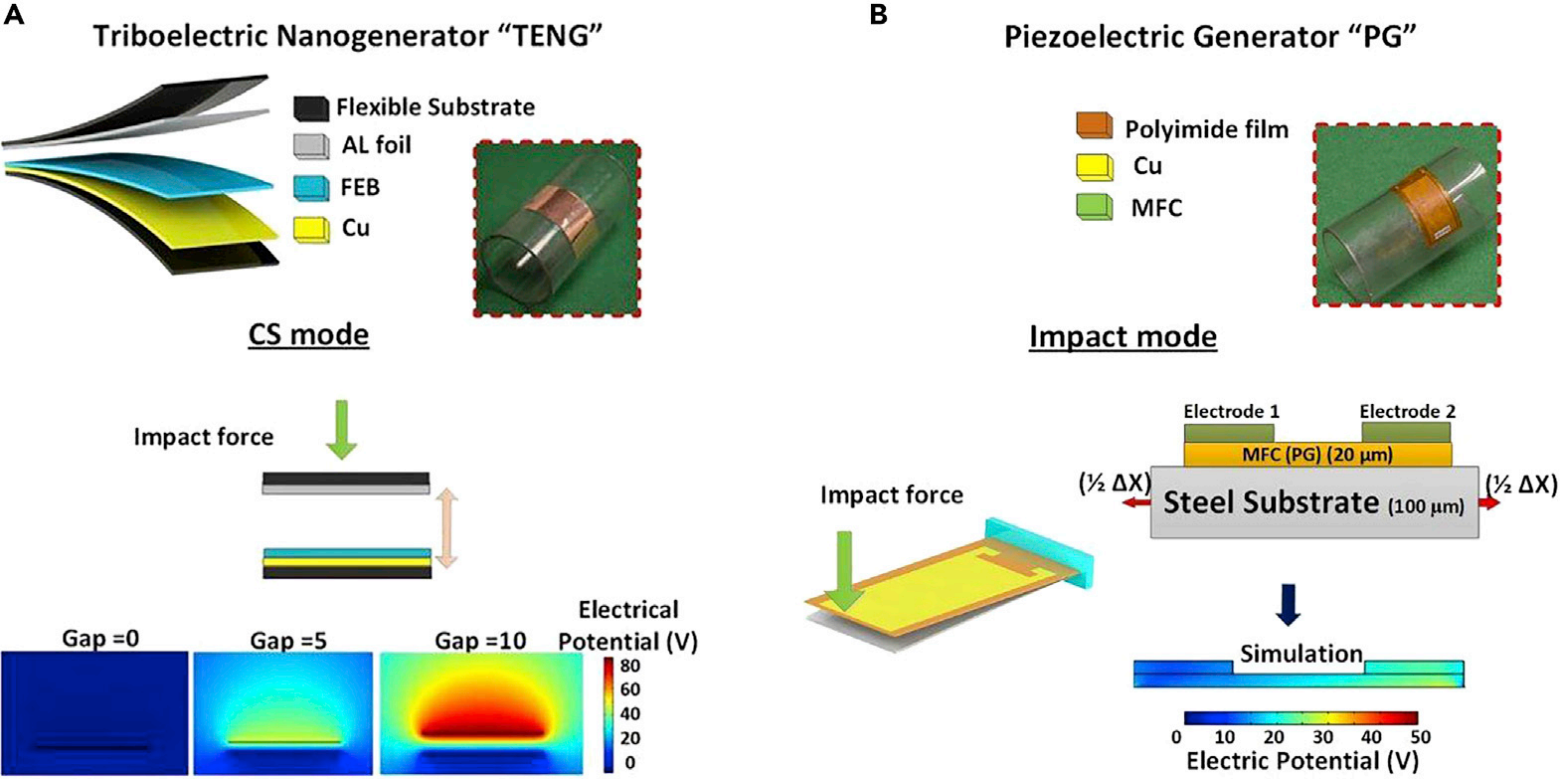
Direct Comparison of the Operation Modes and FEM Modeling of TENG and PG, Respectively Schematic illustration, photograph of the fabricated devices, and EFM modeling for (A) TENG and (B) PG devices.

Figure 1A shows active materials and electrodes used for fabricating the TENG. The CS mode harvests energy by contact separation between the fluorinated ethylene propylene (FEB) layer and aluminum (Al) layer. The size and weight of PGs and TENGs are listed in Methods. For the computational part, we conducted both TENG and PG simulations at the same load conditions. Using the Multiphysics COMSOL software, a simulation was conducted to obtain the electrical potential field of the TENG at separation distances of 0, 5, and 10 mm as shown in Figure 1A. When there is no separation between the FEP and Al layers, the ensuing potential field is approximately zero at the interface. At a separation distance of 10 mm, the CS mode generates an electrical potential up to 80 V. Hence, the electrical potential shows a significant increase with larger separation distances. A computational model was also applied to the PG devices (Park et al., 2017; Jeong et al., 2017b), as shown in Figure 1B. Owing to its remarkable piezoelectric effect, lead zirconate titanate (PZT) ceramic is used in the prototypes of PG devices. This has now been replaced with a Macro Fiber Composite (MFC) in commercial devices as it offers high performance, flexibility, and low cost. The device consists of uniaxial piezoelectric ceramic fibers surrounded by a polymer matrix such as epoxy, which provides protection for the fibers and allows the materials to readily conform to curved surfaces. This unique design combines the power density of piezoceramic materials with the flexibility of polymers. However, a simplified representation of the PG materials is obtained by using PZT as the PG material for simulation, which according to the manufacturer has properties similar to those of MFC (dos Santos Guimarães et al., 2010). The observed piezoelectric field had a consistent distribution of roughly 26 V between adjacent electrodes under impact mode.

**Figure 2:**
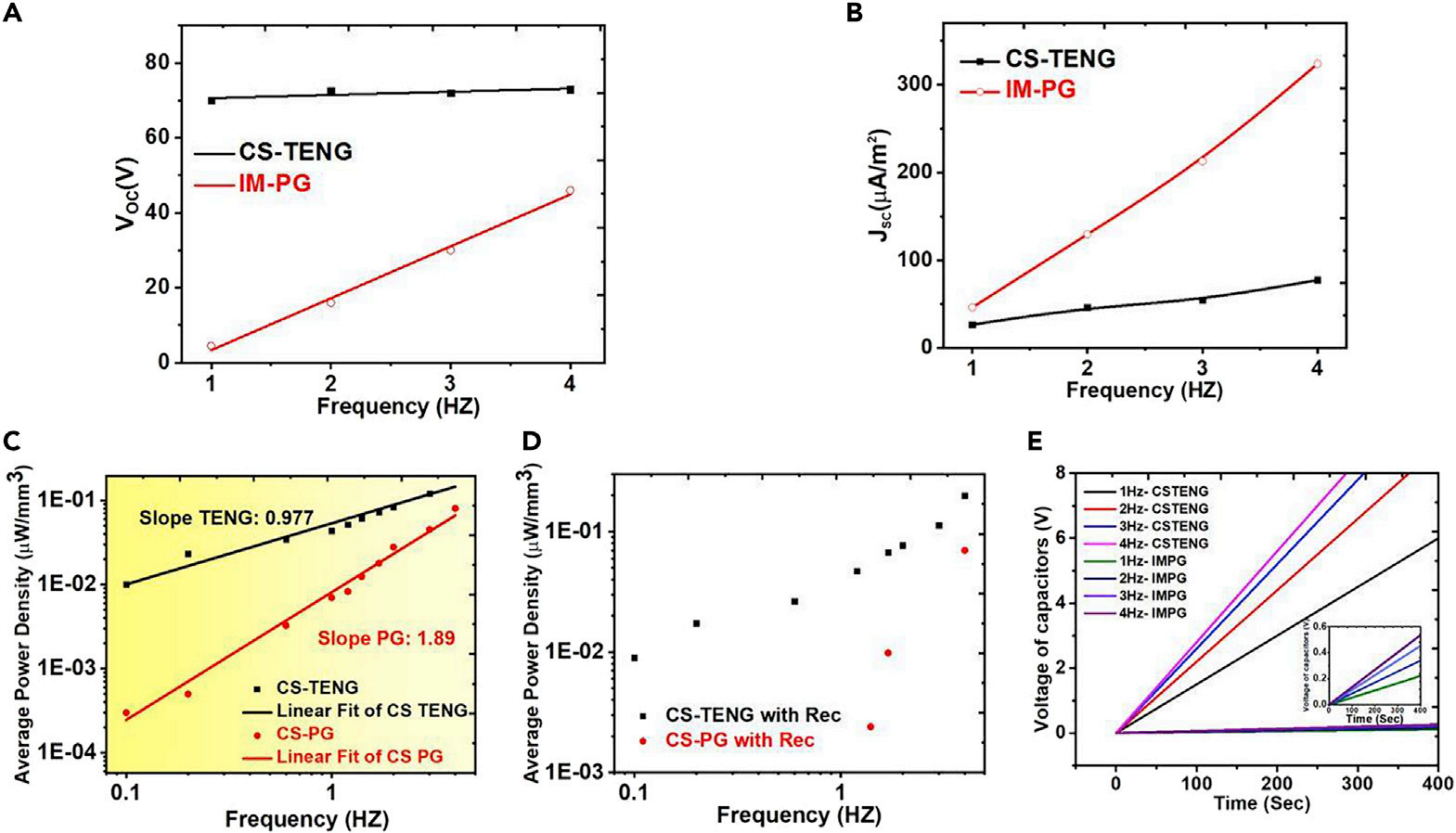
Evaluation of the Electric Properties and Performance of TENG and PG at Low Frequencies (A and B) Open circuit voltage and short circuit current for TENG and PG at different frequencies. (C and D) (C) The average power densities generated at multiple low-frequency values for the two generators. The electrical output performance of the two generators is also shown after connecting to a rectifier for (D) TENG and PG, respectively. Charging performance of a capacitor at multiple operating frequencies is investigated for the two generators. (E) Charging performance of a capacitor at multiple operating frequencies is investigated for the two generators.

Initially, we measured open-circuit voltages ${\text{V}}_{\text{oc}}$ and short-circuit currents ${\text{I}}_{\text{SC}}$ for both devices, while each measurement is conducted at different mechanical load frequencies. Figure 2 summarizes the results, with a prescribed mechanical motion outlined in Methods. According to data in Figure 2A, TENG shows a highly stable voltage output and is almost independent of the applied frequency, whereas the voltage of PGs increases linearly with the applied frequency. Nevertheless, the PG could only deliver around half the voltage of the TENG even when tested at maximum frequency (4 Hz). Based on the working mechanism of piezoelectric energy harvesters, the frequency-dependent behavior of PG output can be easily addressed. When the mechanical input frequency increases, the electron flow through the external circuit and instrument shows shorter time to respond to the piezo-potential change, then this causes the higher current signal, because the total amount of electron flow should be same. Since the voltage signal is the product of the current and circuital resistance (impedance), the voltage also becomes higher correspondingly. This linear relationship causes PG generators to work only under constant periodic loads and limits their use in a multi-frequency environment (Gu et al., 2012). According to the previous reports, the voltage level can be saturated at a certain input frequency owing to the device structures, measurement conditions, and so on (Gu et al., 2012; Yang et al., 2017). For our comparison study, the measurements were conducted in off-resonance and tapping modes (<5 Hz), showing the frequency-dependent relationship of PG devices (Gu et al., 2012). Furthermore, it is known that PGs generally deliver higher current values than TENGs, which is also true here as can be seen in Figure 2B. However, the current shows direct proportionality with frequency.

Next, we experimentally demonstrated the average power density (per unit volume) and its correlation with the operating frequency. Figure 2C compares the performance of TENG and PG systems, respectively. Given the wide range of frequencies and power, the data are presented on a logarithmic scale, where the TENG had almost twice the power density of the PG system at the maximum operating frequency for both modes. The slope of the fitted data has been calculated to manifest the consistency of power generation at multiple frequency values. It can be observed that TENGs had a much lower energy density variation than PGs, as lined by the calculated slope. The generated energy of PGs shows a strong dependence on the input frequency, whereas the TENG exhibits stable performance and is less dependent on the applied frequency. As discussed previously, the voltage of PG is proportional to the triggering frequency ω. This corresponds well to the broadband frequency operation of TENGs to sustain operations at multiple frequency levels, as opposed to variable PG outputs.

Figure 2D shows the power density results of each generator after connecting the rectifier. It can be observed that the TENG was able to produce electrical output with frequencies as small as 0.2 Hz. Moreover, this electrical output was still maintained for multiple frequencies after connecting the rectifier. However, PG was only able to produce electrical output at a minimum frequency value of 1 Hz (one order of magnitude higher than the minimum operating frequency of TENG) when connected with a rectifier. This limits the various works of PG harvesters for environments with extremely low frequencies, especially when powering up an electrical component or charging up a battery. Because most of the output power disappears after connecting to a rectifier, the PG harvesters might be impractical for generating or storing sustainable power combined with a rectifying circuit. To ascertain such observations, Figure 2E shows the voltage of charging a capacitor using the two harvesters, respectively, and the time it takes to charge the capacitor at multiple frequency values. Consequently, the previous results show that most of the charge density is omitted for the PG harvesters and the obtained charge is only about half of that reached by TENG-based harvesters at only the maximum frequency. That corresponds well with the current capacitor's voltage results as the PG harvesters are unable to charge the capacitor to a large voltage within the time span of 400 s, reaching a maximum of 0.6 V for PG (see inset in Figure 2E).

**Figure 3:**
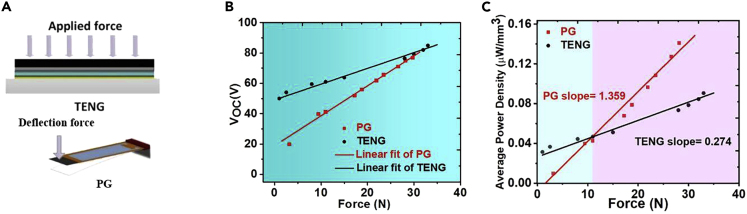
Electrical Performance of TENG and PG as a Function of the Applied Force (A) Schematic illustration of the TENG and PG under different applied forces. (B) The measured open-circuit voltages at different applied forces. (C) The average power densities at different applied forces.

Next, we investigate the effect of load magnitude on the energy harvesting performance for the two generator types as portrayed in Figure 3A. Figure 3B shows the open-circuit voltage results, for the two generators with a linear fit of the data. The TENG experienced much higher voltages at lower values of applied loads, where the PG performance approaches that of the TENG as the load magnitude increases. To verify the power performance of the two harvesters, the generated power was calculated for the two cases and compared in Figure 3C. At very low regions, the TENG performed well at a load range of approximately 10 N and below. The 10 N mark represents a break-even point, above which the PG starts to outperform TENGs thanks to the superior power performance of the PG device, which seems to increase more rapidly with the applied force, as shown in Figure 3C.

It is worth noting that the power performance for the two devices under different loads (Figure 3) follows a different trend from that when tested as a function of the operating frequency of the source (Figure 2). The main difference between the two generators regarding the loading profile is that the PGs were exposed to a point load at the end of the cantilever-like structure, whereas TENGs experienced a distributed load to cover its contact area. Therefore, the maximum deflection of a cantilever beam due to a point load is directly proportional to the load magnitude (Takács, 2012). As such, increased deflection of the PG will create an enhanced electrical output due to the piezoelectric effect. As for, TENGs has experienced an increased slope of average power with elevated mechanical loads. Low distributed loads in a contact-separation motion may not ensure full contact area between the two triboelectric layers. However, additional loads would enhance the contact surface (i.e., increase the total contact area), which would enhance the electrical output from the TENGs and their surface charges.

## Discussion

**Figure 4:**
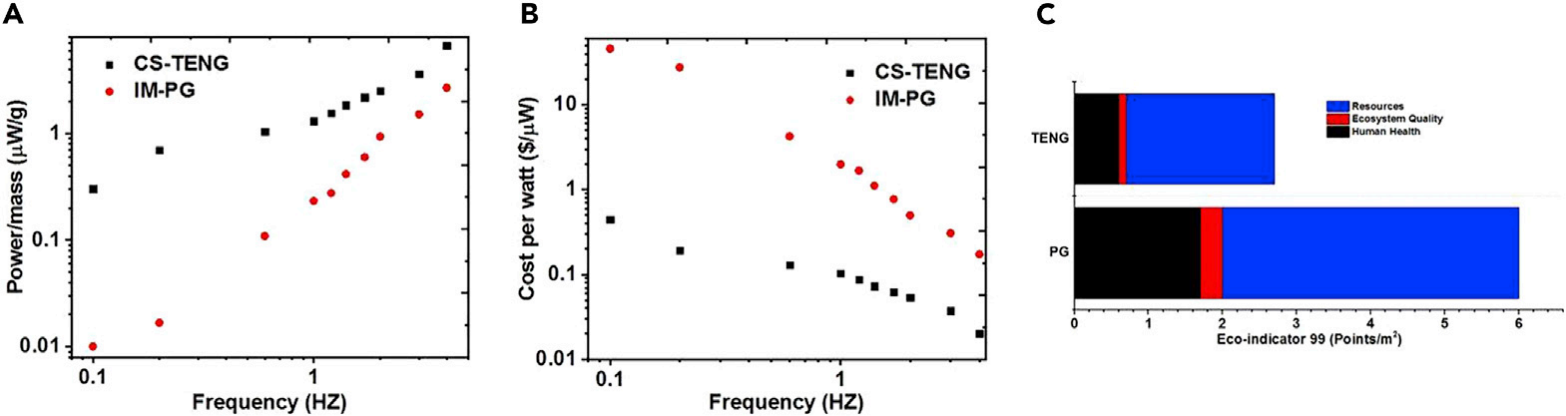
Specific Power, Cost per Watt, and Techno-Economic Analysis of TENG and PG (A) The average power-to-mass ratio of PG and TENG. (B) The cost per watt of PG and TENG. (C) Eco-indicator 99 results for 1 m^2^ of each mode. The data for TENG is extracted from the study of Ahmed et al., 2017. The data for PG is extracted from the study of Ibn-Mohammed et al. (2016).

We conducted a comparative analysis of the harvesters' weight and economic performances relative to the produced power. Figures 4A and 4B show the specific power (power normalized to the weight of the device) generated by both energy harvesters. Both TENG and PG exhibit adequate energy harvesting capabilities with a slight advantage to TENGs regarding power generation per unit mass. The specific power experiences an increase with increasing frequency. On the other hand, there is a considerable difference in cost per watt between the two harvesters at extremely low-frequency values (around 1 Hz). At this frequency, the cost of PGs is higher than that of TENGs. However, the cost of PG power goes down very rapidly as the frequency increases. In contrast, the cost of TENGs is relatively constant, making it highly efficient regarding multi-frequency operation at low-frequency scales.

As energy harvesting technologies, the two mechanical generator types should be investigated within a cost-metric context to ascertain their performance regarding the cost of materials, fabrication, and their environmental profiles. Here, we discuss the techno-economic of the TENG (Ahmed et al., 2017) and PG (Ibn-Mohammed et al., 2016), where a chosen metric was assigned with its data presented in Figure 4C. The metric chosen was the Eco-indicator 99, which provides an insight into whether the technologies constitute any significant environmental limitations. The three categories for this comparison were ecosystem quality, resources, and human health. In all three categories, PGs showed higher Eco-indicator 99 (environmental impact) values than TENGs, with a more visible difference coming from resources and human health. The comparative study had shown a general preference for TENGs over PGs when operating in the low frequency/force amplitude regions. This excellent performance opens up the door for a wide range of self-powered electronics for wearable and wireless sensing applications. Such wearable devices would require a minimal effort to operate, charge, and/or activate embedded electronics by mere human motion and surrounding environmental conditions.

In summary, this work presents a systematic comparative study between PGs and TENGs as potential energy harvesting devices operating in low-frequency conditions. In general, PGs exhibit low voltage and high current output, with both ${\text{V}}_{\text{OC}}$ and ${\text{I}}_{\text{SC}}$ having a visible proportionality with the operating frequency. On the other hand, measurements show that TENGs do experience high voltage outputs with minimal proportionality to the operating frequency and low current output. These attributes enable TENGs to demonstrate much higher power density compared with PGs along the low-frequency range (below 1 Hz). At these frequency levels, the extremely low voltage values obtained by PGs limit their applications in powering up electronic devices and energy storage units with rectifiers. Moreover, the energy performance against the load magnitude confirmed that the TENGs are superior at lower mechanical loads values. However, break-even analysis shows that PGs are better at higher values within the portrayed load magnitude range. Finally, the work reveals a considerable opportunity for TENGs in wearable, self-powered electronics powered by low-frequency/amplitude mechanical motions associated with human movements.

### Limitations of Study

We provide a detail comparison of TENG and PG in low frequency, which is helpful for researchers. However, theoretical investigation can be conducted to get in-depth understanding for both generators under different modes. In addition, different device modes such as, cantilever mode can be used to study different effects on the generator's performance.

### Resource Availability

#### Lead Contact

Abdelsalam Ahmed.

#### Materials Availability

This study did not generate new unique reagents.

#### Data and Code Availability

We do not have any code and upon request we can provide the original data.

## Methods

All methods can be found in the accompanying Transparent Methods supplemental file.

## Supplemental Information

Document S1. Transparent Methods
