# Supplementary material for: Triboelectric Nanogenerator versus Piezoelectric Generator at Low Frequency (<4 Hz): A Quantitative Comparison
Source: iScience. 2020 Jun 20;23(7):101286. doi: 10.1016/j.isci.2020.101286 (PMC7334414; doi:10.1016/j.isci.2020.101286)
Supplement: Document S1. Transparent Methods [file mmc1.pdf]

iScience, Volume *23*

Supplemental Information

Triboelectric Nanogenerator versus Piezoelectric Generator at Low Frequency (<4 Hz): A Quantitative Comparison

Abdelsalam Ahmed, Islam Hassan, Ahmed S. Helal, Vitor Sencadas, Ali Radhi, Chang Kyu Jeong, and Maher F. El-Kady

Fabrication of the TENG: A 100 µm thick FEP film (dimensions 18 × 40 mm) was coated with a layer of 200 nm thick Cu electrodes using physical vapor deposition (PVD500) before being attached to the substrate. This was brought in contact with the other electrode, a 100 µm thick Al foil with these dimensions 18 x 60 mm. This stack was sandwiched between two pieces of polyvinyl chloride (PVC, dimension 18 × 40 mm and thickness 0.5 mm) to provide the physical support for the device. The overall weight of the stack was measured to be 3.7g.

Fabrication of PG: The PG device was made by winding a commercial Macro Fiber Composite MFC (28 × 14 mm, model M-2814-P1 from Smart-Material, Corp) around a 0.1 mm thick steel sheet (18 × 40 mm) and is approximately 5 g in weight. The chosen MFC consists of rectangular piezoceramic rods sandwiched between two adhesive layers with an $d_{33} = 460$ pC/N (Smart-Material.com, 2020). In this case, the ceramic is lead zirconate titanate (PZT) with the chemical formula ($Pb[Zr_{(x)}Ti_{(1-x)}]O_3$), whereas epoxy was used as the connecting polymer matrix. To complete the PG device, the MFC layer is attached to a polyimide film with copper back contact electrodes that are typically applied in an interdigitated pattern.

Electromechanical Measurements: A three-dimensional positioner was used to deploy the nanogenerator (TENG or PG) in the vertical orientation. A force sensor was then attached to the end of a linear motor, enabling precise measurements. In this setup, the output current and voltage data were collected by a voltage preamplifier (Keithley 6514 System Electrometer). We implemented LabVIEW software as a platform for real-time data acquisition and control analysis. In the course of these experiments, different levels of external loads were tested during power measurements. This experiment utilized a linear motor to produce periodic loads with several magnitudes and frequencies. Starting from time t = 0), the motion profile can be expressed as shown below:

$$X = \begin{cases} 8X_{max}f^2t^2 & ; 0 \le t \le \frac{1}{4f} \\ 8X_{max}ft - 8X_{max}f^2t^2 - X_{max} & ; \frac{1}{4f} \le t \le \frac{3}{4f} \\ 8X_{max}f^2(\frac{1}{f} - t)^2 & ; \frac{3}{4f} \le t \le \frac{1}{4f} \end{cases}$$

Simulation and modeling:

TENG: The multiphysics study for the TENG was conducted by COMSOL. The potential field results were obtained between the Al and Cu electrodes, where the model was set up with the original device dimensions with deposited Cu on the FEP film. The film has dimensions of 18 × 40 mm. The floating potential terminal was assigned to the Al electrode with the Cu electrode connected to the ground. Positive and negative triboelectric charge densities were allocated at tens of $\mu C/m^2$ to the Al and FEP layer, respectively.

PG: The model was set with a 20 µm piezoelectric layer and conductive electrodes with PZT as the material model for the piezoelectric simulation [8]. The electrodes were separated by a 200 µm gap. A Young's modulus of 54.05 GPa was selected for the finite element model along with a piezoelectric charge constant d_{33} of 440 pC/N. The active area density ρ and the dielectric constant K_T were set as 5.44 g/cm^3 and 1950, respectively. [9] The applied strain was obtained from the bending radius and the thickness of the multilayer substrate, which is expressed as $\varepsilon = \delta / r$ [10]. This formula is derived from the neutral plane distance from the top surface of the MFC film δ, and the PG bending radius r. This applies to flexible MFC devices containing a plastic surface with an epoxy passivation layer. In the PG simulation, figure 1, the results show the potential field between the electrodes only. Also, the open-circuit voltage contour was outputted without any external loads. The TENG simulation shows the field over its overall dimensions. Identical boundary conditions were utilized for both simulations.
